# Improved linkage analysis of Quantitative Trait Loci using bulk segregants unveils a novel determinant of high ethanol tolerance in yeast

**DOI:** 10.1186/1471-2164-15-207

**Published:** 2014-03-19

**Authors:** Jorge Duitama, Aminael Sánchez-Rodríguez, Annelies Goovaerts, Sergio Pulido-Tamayo, Georg Hubmann, María R Foulquié-Moreno, Johan M Thevelein, Kevin J Verstrepen, Kathleen Marchal

**Affiliations:** 1 VIB Laboratory of Systems Biology & Laboratory for Genetics and Genomics, Centre of Microbial and Plant Genetics, KU Leuven, Gaston Geenslaan 1, Leuven B-3001, Belgium; 2 Department of Microbial and Molecular Systems, Centre of Microbial and Plant Genetics, KU Leuven, Kasteelpark Arenberg 20, Leuven B-3001, Belgium; 3 VIB Department of Molecular Microbiology & Laboratory of Molecular Cell Biology, Institute of Botany and Microbiology, KU Leuven, Kasteelpark Arenberg 31, Leuven B-3001, Belgium; 4 Department of Plant Biotechnology and Bioinformatics, Ghent University, Ghent 9052, Belgium; 5 Department of Information Technology, Ghent University, IMinds, VIB, Gent 9052, Belgium

## Abstract

**Background:**

Bulk segregant analysis (BSA) coupled to high throughput sequencing is a powerful method to map genomic regions related with phenotypes of interest. It relies on crossing two parents, one inferior and one superior for a trait of interest. Segregants displaying the trait of the superior parent are pooled, the DNA extracted and sequenced. Genomic regions linked to the trait of interest are identified by searching the pool for overrepresented alleles that normally originate from the superior parent. BSA data analysis is non-trivial due to sequencing, alignment and screening errors.

**Results:**

To increase the power of the BSA technology and obtain a better distinction between spuriously and truly linked regions, we developed EXPLoRA (EXtraction of over-rePresented aLleles in BSA), an algorithm for BSA data analysis that explicitly models the dependency between neighboring marker sites by exploiting the properties of linkage disequilibrium through a Hidden Markov Model (HMM).

Reanalyzing a BSA dataset for high ethanol tolerance in yeast allowed reliably identifying QTLs linked to this phenotype that could not be identified with statistical significance in the original study. Experimental validation of one of the least pronounced linked regions, by identifying its causative gene *VPS70*, confirmed the potential of our method.

**Conclusions:**

EXPLoRA has a performance at least as good as the state-of-the-art and it is robust even at low signal to noise ratio’s i.e. when the true linkage signal is diluted by sampling, screening errors or when few segregants are available.

## Background

**Figure 1:**
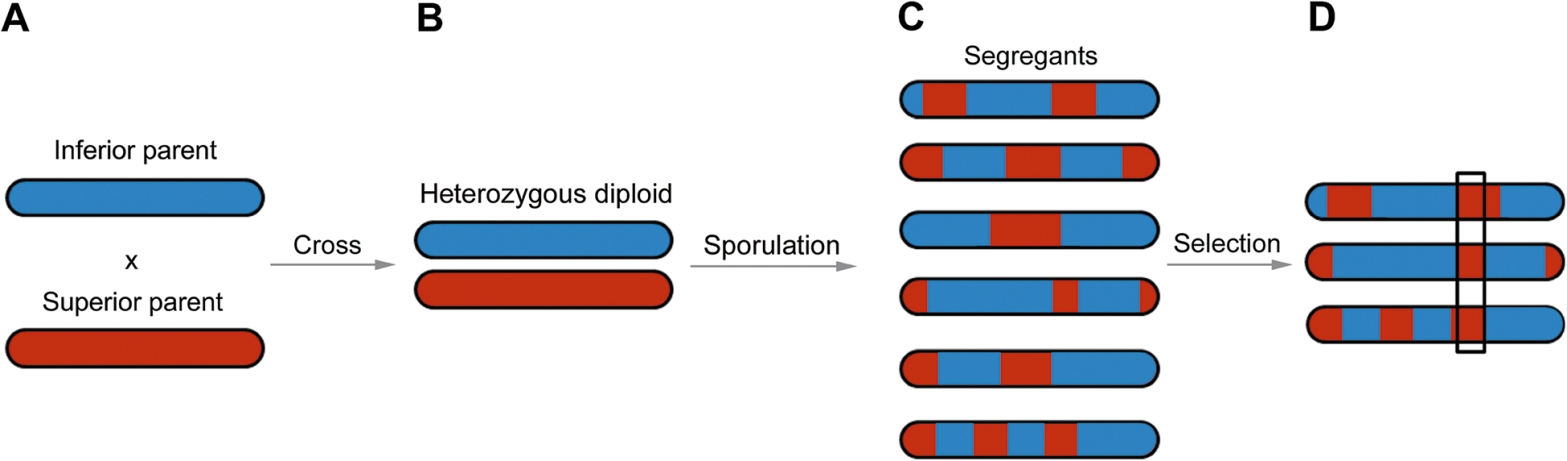
**Bulk segregant analysis for mapping genomic regions linked to a phenotype of interest in yeast. A**: A parent displaying the phenotypic trait of interest (superior parent) is crossed with a reference strain lacking the trait (inferior parent). **B**: The resulting heterozygous diploid strain is then sporulated to generate haploid segregants. **C**: Segregating offspring carry a mosaic of genetic material derived from both parents (red and blue segments) due to the recombination events in meiosis. After phenotyping, the subset of segregants displaying the trait of the superior parent is selected. **D**: Genomic DNA extracted from the pooled selected segregants is submitted to whole-genome sequence analysis. Polymorphic genomic regions (marker sites) are identified that allow distinguishing between the parental variants. Counting for each marker site how many variants originate from the superior versus the inferior parent allows determining the variant frequency in the pool for each marker site. Regions linked to the phenotype of interest are expected to originate predominantly from the superior parent (black boxed region). The principle of BSA with diploid organisms is similar, but usually inbred (homozygous) lines are used as parents and two generations are needed to observe segregation of the phenotype.

Bulk segregant analysis (BSA) is an elegant method that allows simultaneous identification of genetic loci that contribute to a specific trait or phenotype (for a review see Liti and Schacherer [1] and references therein). Recently, BSA has been coupled to high throughput sequencing methods (for a review see Swinnen et al. [2] and references therein). In such a BSA set up, an individual displaying a phenotype of interest (superior parent) is crossed with a reference (inferior) parent lacking this phenotype to generate a population of segregants. Subsequently, the segregants are screened to identify a subset displaying the phenotype of interest. These selected individuals are pooled together (here referred to as the “selected pool”), and the genomic DNA of the pool isolated. High-coverage sequencing of this pooled genomic DNA allows identifying for each polymorphic genomic site (referred to as genetic marker sites) the relative frequency of the two (superior and inferior) parental variants in the pool. Variant frequencies of these SNPs should theoretically be 50% for either parent variant, except for those regions that are genetically linked to the phenotype of interest. At those regions, often referred to as Quantitative Trait Loci (QTLs), the causative allele from the superior parent will be over-represented. The corresponding allele of the inferior parent will be under-represented. Figure 1 shows a schematic representation of this approach, which has been successfully applied amongst others in *Saccharomyces cerevisiae* for high ethanol tolerance [3], impaired vacuole inheritance [4], xylose utilization [5], heat tolerance [6], variation in colony morphology [7], tolerance to 23 different ecologically relevant environments [8] and 17 chemical resistance traits [9]; in *Zea mays* for drought resistance [10]; in *Arabidopsis thaliana* for growth defects [11] and cell wall composition [12]; in *Oryza sativa* to find agronomically important loci [13] and in *Danio rerio* to study developmental mutants [14].

Theoretically, for any marker site not linked to the phenotype of interest, the alleles in the pool of segregants should be inherited in nearly equal proportions (50%) from either parent. A statistical test (e.g., Birkeland et al. [4], Swinnen et al. [3]) can be applied for each genetic marker separately to assess the extent to which the variant frequency at the marker site deviates from the expected inheritance probability of 50%. Hence, the power of QTL mapping by BSA depends on the size of the initial population of segregants, the size of the selected pool and the strength on the phenotype (QTL effect). However, the sequencing procedure can compromise the QTL-mapping power: the sequencing coverage should at least be equal to the number of segregants to ensure information retrieval from all segregants [7]. When the coverage is too low, variant frequencies at marker sites will deviate significantly from the theoretical 50% in phenotype-neutral regions due to sampling error. In addition, errors introduced during library preparation, sequencing, read alignment and SNP calling can also cause bias in variant frequency and result in falsely linked regions (regions not truly related to the phenotype). As a result, in reality, spurious deviations of the observed variant frequencies from the theoretical 50% at marker sites will occur due to different sources of experimental error.

To increase the power of QTL mapping by BSA the properties of linkage disequilibrium can be exploited. Linkage disequilibrium (LD) arises because proximal marker sites are co-inherited [15]: in a BSA set up, a causative mutation will thus always be embedded in a larger region of marker sites that all display a deviation from the theoretical 50% inheritance of either parental variant. The extent of the deviation decreases with the distance to the causative mutation and depends on the resolution of the BSA. Linkage disequilibrium produces deviations of variant counts towards the superior variant, not only at the genetic marker site(s) causative to the phenotype of interest, but also in genetic marker sites closely located to these causative marker sites.

State-of-the-art BSA methods exploit LD to increase the power of BSA analysis but they differ in the way LD is modeled. A first set of methods model LD in a mere data driven way: relative variant frequencies are fitted robustly fit using a sliding window based strategy followed by different smoothing functions [3,7,9,11,12]. More recently, Edwards and Gifford [16] developed a Bayesian network called MULTIPOOL to estimate the probability of linkage for each site and Leshchiner et al. [14] developed an HMM tailored to perform fine mapping of causative sites in mutagenesis experiments.

We developed a Hidden Markov Model (HMM) called EXPLoRA that explicitly models the effects of linkage disequilibrium to explain the dependencies between neighboring variant frequencies in the observed data. In contrast with other methods, EXPLoRA models the relationship between a genomic variant and the phenotype of interest as a hidden state and use beta-binomial distributions to calculate emission probabilities of the observed data. Tests on simulated data show that EXPLoRA outperforms currently available state-of-the-art algorithms especially in cases where only a limited number of selected segregants can be produced. To further assess the performance of EXPLoRA we analyzed a recently published dataset, described in Swinnen et al. [3], in which three different pools of yeast segregants were used, two of which were selected for tolerance to a different high level of ethanol and one which was used as unselected control pool. Upon re-analysis of the data of Swinnen et al. [3] with our HMM model, we were able to identify reliably QTLs linked to ethanol tolerance that could not be identified with statistical significance in the original study [3]. An open source java implementation of EXPLoRA, useful for external use and independent validation is available at: http://bioinformatics.intec.ugent.be/kmarchal/Supplementary_Information_Duitama_2013/.

## Methods

### EXPLoRA method

**Figure 2:**
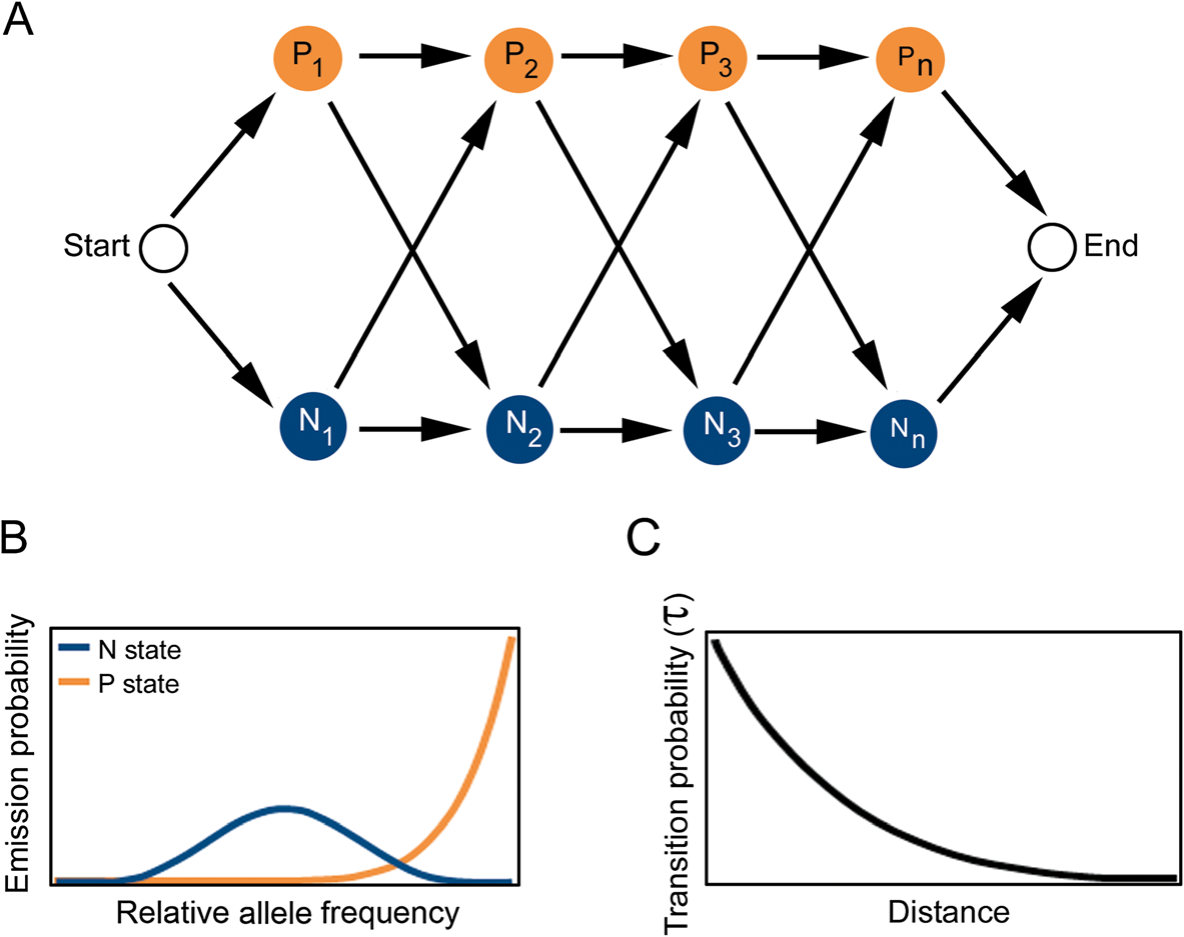
**Hidden Markov Model used to predict genomic regions linked to the phenotype of interest. A**: each marker site is modeled to be in a neutral state (N-state, blue circles) or in a state of being linked to the phenotype of interest (P-state, orange circles) based on its observed relative variant frequency in the pool of segregants. **B**: emission probabilities for respectively the neutral (blue curve) and the phenotype-linked states (orange line) as a function of the relative variant frequencies, modeled by a beta-binomial distribution with respective parameters α and β. **C**: transition probability as a function of the physical distance between neighboring marker sites.

EXPLoRA is a Hidden Markov Model (HMM) which has per marker site two emission probabilities that model respectively that the variants in the pool at the marker site originate from the superior parent (P-state) or to an equal extent from either parent (N-state). The effect of linkage disequilibrium is modeled by the transition probabilities τ between two neighboring marker sites. The transition probability τ models the chance that a neighboring site remains in the same state as its preceding site state. Its distribution is described by a negative exponential model as a function of the recombination rate and the physical distance between neighboring marker sites [17] (Figure 2C).

Given a random state N_i_ or P_i_ at a marker site ‘i’ , the transition probabilities to the states N_i_ + 1 or P_i_ + 1 for the neighboring marker site ‘i + 1’ are given by:

$$\tau_{N_{i}\to N_{i+1}=1-e^{-rl_{i}}}$$

or

$$\tau_{P_{i}\to P_{i+1}=1-e^{-rl_{i}}}s$$

where *l*_*i*_ is the physical distance between the marker sites *i* and *i* + 1 and *r* is a recombination rate, which is determined by the average number of crossing-overs occurring during meiosis over a given distance in a chromosome. The default level of *r* was fixed at 3.5 × 10^-6^, based on the estimations derived by Ruderfer et al*.*[17].

Each state in the model emits a random variable *n*_A_, corresponding to the number of variant counts at a given marker site originating from the superior parent. *n*_A_ is described by a beta binomial distribution, which allows capturing different emission probabilities in phenotype-linked versus neutral states by choosing different α and β parameters for their corresponding distributions (Figure 2B). We modeled all neutral states with the same parameters α_N_ and β_N_, and all phenotype-linked states with the same parameters α_P_ and β_P_.

Given the observed total variant count and the variant counts that originate from the superior parent at each marker site (*D*) and fixed values for the parameters α_N_, β_N_, α_P_, β_P_, and τ, we can calculate the posterior probability of each state in the HMM with a standard forward-backward algorithm [17]. For each marker site, we then estimate its probability to be linked to the phenotype of interest as the normalized probability P(P_i_ | *D*) / (P(P_i_ | *D*) + P(N_i_ | *D*)).

Since most of the genomic regions are supposed to be neutral with respect to the phenotype of interest, the parameters α_N_ and β_N_ of the emission probabilities in the neutral state can be estimated directly from the observed variant frequencies. To this end, we implemented a two-step process in which we first assume that most of the genomic regions are phenotype-neutral. We estimate with the method of moments the most likely values of α_N_ and β_N_ given the variant frequencies at each marker site. Then in a second step we identify the marker sites linked to the phenotype of interest using the model, and we estimate again α_N_ and β_N_ leaving out the marker sites identified to be linked to the phenotype.

### Simulated data

To assess the robustness of EXPLoRA we conducted simulations as follows: an artificial chromosome of length 750 kbp with random polymorphic sites was simulated. A single site was randomly chosen to be causative. For each simulation we defined in advance a proportion of segregants in the selected pool with the causative site (referred to as the PSC). This proportion is used to construct a selected pool as follows: each segregant originates by randomly combining both parental alleles. So each segregant has a probability of 50% to contain the causal variant. Each segregant with the causal variant has a probability equal to the PSC to be present in the final pool whereas a segregant without the causal variant has a probability of 1-PSC. Segregants are added to the pool until the final number of selected segregants is reached (*n*). By defining in the simulations the ‘noise level’ as the PSC we avoid to make any assumptions on the cause of the ‘noise level’ (which can both be attributed to an incomplete QTL effect or to a difficult selection procedure of the selected segregants) and the subsequent choice of an explicit model to describe the ‘QTL effect’ of the segregants. It is important to note that in this simulation set up, a higher number of segregants (*n*) does not increase the noise level (as is the case for simulations that rely on an explicit phenotypic model [7]). The effect of *n* only affects the results through its effect on the statistical power (if applicable) or because at low values of *n* the relative impact of a sampling error will be higher. Pools of selected segregants of size *n* were created by recombining the parental strains at a constant recombination rate of 0.37 centimorgans (cM) per kilobase, which is the average value for a yeast chromosome [18]. Sequences of the selected pools were simulated at variable coverage (*c*) with a constant sequencing error rate of 0.01 (corresponding to the reported Illumina sequencing error [19]). A total of 100 datasets were created for each tested combination of simulation parameters.

### Performance analysis

To test the effect of the parameters on the performance of EXPLoRA we used the fixed simulation parameters mentioned above and the following variable ones: *n* = 30, *c* = 200. The PSC was varied from 0.6 to 0.95 and the number of polymorphic sites (marker sites) was changed from 10 to 10000. The α_P_/β_P_ ratio was varied from 5 to 40 and the assumed recombination rate (*r*) was changed from 3.5 x 10^-8^ to 3.5 x 10^-3^. For each setting we report the recovery rate (i.e. the capacity to retrieve the region in which the causal site is embedded), the size of the linked region containing the position of the true causal site and the number of false positive linked regions.

### Comparison with state-of-the-art

To perform a comparison with Magwene et al. [7] and MULTIPOOL [16] we used the fixed simulation parameters mentioned above and the following variable ones: 2 500 random polymorphic sites of which a single site was randomly chosen to be causative. Two noise scenarios are presented: Low Noise with a PSC of 0.95 indicating that around 95% of the selected segregants contained the causative allele of the superior parent, and High Noise scenario with a PSC of 0.85. Pools with an increasing number of segregants (*n* = 5, 10, 20, 30, 200, 500, 1000 and 2000) were simulated. Sequencing of the selected pools was simulated at variable coverage (*c* = 30, 50, 100, 200, 500 and 1000).

A standalone version of the method described by Magwene et al. [7] was obtained from the authors and MULTIPOOL [16] was downloaded from http://cgs.csail.mit.edu/multipool/. For the purpose of comparison all tools were run on the simulated data (see above). To assess recovery rate we measured for each method the number of times that the region in which the causative site was embedded was found to be significantly linked divided by 100 (the number of repeats for each experimental setup). For EXPLoRA a marker is significantly identified if the posterior probability assigned to the marker is larger than 0.95. For the method of Magwene et al. [7] we calculated for each experiment the null distribution of the G' score using the non-parametric method described by the authors. Based on this null distribution, we calculated a p-value for each marker, also following the method described in [7]. A marker is significantly linked with the phenotype if its p-value passes correction for multiple testing at a 0.05 significance level [7,20]. Two types of corrections for multiple testing (simple and robust) were applied [7]. For MULTIPOOL a marker is significantly linked if its LOD (log10 likelihood ratio) score falls within a 90% confidence interval [16].

Specificity is measured using two metrics: the size of the linked region at the causal position and the number of false positive linked regions found. We ran the method of Magwene et al. with a default genetic window size of 30 cM, as recommended by the authors [7]. For EXPLoRA we fixed the α_P_/β_P_ ratio at 15 which gives the best tradeoff between the recovery rate and the size of the predicted regions. MULTIPOOL was run with the default discrete block size of 100 bp [16].

### Real dataset

To test our method, we used the dataset reported by Swinnen et al. [3]. In their work, a segregant, VR1-5B (superior parent) from a Brazilian bioethanol production strain VR1 was crossed with the BY4741 lab strain (inferior parent). A total of 136 segregants tolerant to 16% ethanol and out of these, 31 segregants also tolerant to 17% ethanol, were pooled. DNA of the pools and also of the VR1-5B parental strain was extracted and sequenced using Illumina technology (100 bp reads) [3]. A total of 131 unselected segregants from the same cross were also pooled and sequenced as control experiment (unselected pool).

Marker sites were identified as follows: the yeast S288c reference genome (3 Feb. 2011 release) available in the Saccharomyces Genome Database (http://www.yeastgenome.org) was used as reference. All reads from the parental strain VR1-5B were mapped to the reference sequence using bowtie2 [21]. We used the -a option to retain as many good alignments as possible for each read. Over 93% of the reads from VR1-5B, 84% and 86% of the reads from the pools of segregants under selection, and 98% of the reads from the pool of unselected segregants could be mapped to the latest reference genome. We ignored the last 25 bp of each read from the VR1-5B strain and the two pools of selected segregants based on the base calling error rate estimated from unique alignments.

SNPs and small indels between the two parents VR1-5B and S288c (the reference sequence) were identified with the SNVQ algorithm [22]. We filtered out predicted variants with genotype quality scores lower than 40, falling into annotated repetitive regions (i.e., transposons, telomeres, centromeres), or falling into duplicated regions predicted either by reads with multiple alignments or by the CNVnator algorithm [23]. Finally, we filtered out predicted variants located less than 30 bp from each other to avoid undesired local errors due to misaligned reads. We obtained 25,972 SNPs and 1,429 indels which were used for analysis of segregant pools.

To identify the relative variant frequencies in the pools of segregants at marker sites, we implemented a custom script to count at each marker site the number of read alignments that support the variant originating from the superior parent (VR1-5B) and the total number of alignments. Within each pool variants with read coverage less than 20 or over 100 were ignored. We retained 26,913 variants for the 16% pool, 26,865 variants for the 17% pool, and 24553 variants for the pool of unselected segregants.

### Experimental validation

Experimental verification of QTL2 on chromosome X was based on determining for a selected set of marker sites in this region, the number of times individual segregants selected for high ethanol tolerance displayed the variant originating from the superior parent (relative variant frequency in individual segregants) [3]. Relative variant frequencies in individual segregants were used to calculate the posterior probability of each marker site to be linked to the phenotype of interest using an exact binomial test with a confidence level of 95% and correction for multiple testing by a false discovery rate (FDR) control according to Benjamini and Yekutieli [20]. Ethanol tolerance assays and reciprocal hemizygosity analysis were carried out as described previously [3].

## Results

### Development of EXPLoRA, a HMM for the analysis of BSA data

As indicated above, BSA is the first step towards finding sequence variations (also referred to as “alleles”, “variants”) that cause a given phenotype. Causative sequence variations originating from the superior parent are expected to be over-represented in the selected segregant pool. Due to linkage disequilibrium (LD), other variants at marker sites that surround the causative site will also be over-represented in the selected pool. LD thus limits the resolution of the BSA analysis towards identifying the region in which the true causal site is embedded rather than the true causal site. However, this dependency between neighboring sites (LD) can be exploited to increase the power of the statistical linkage of the identified loci to the phenotype of interest by filter out spuriously linked regions. To exploit the information contained in the dependency between neighboring marker sites, we developed a Hidden Markov Model (HMM) called EXPLoRA (Figure 2). EXPLoRA explicitly models the effect of linkage disequilibrium to explain the dependencies between neighboring sites in the data. EXPLoRA models for each marker site, two possible states: one state (P-state) expresses that the variants in the pool at that marker site originate predominantly (but not always in all segregants) from the superior parent and are thus linked to the phenotype of interest. A second state (N-state) models that the variants in the pool at a given marker site originate to an equal extent from either parent, in which case the marker site is assumed to be located in a neutral region not linked to the phenotype of interest. The effect of linkage disequilibrium is modeled by the transition probabilities τ between two neighboring marker sites. The transition probability τ models the chance that a neighboring site remains in the same state as its preceding site state. Its distribution is described by a negative exponential model as a function of the recombination rate r and the physical distance between neighboring marker sites [17] (Figure 2C and Materials and methods). The probability to change states upon transition from one marker site to its direct neighboring marker site (from a neutral N-state to a phenotype-linked P-state or vice versa) is then described by 1-τ and takes into account the true distance between them (i.e. no distance binning is involved). The model captures the fact that marker sites located in each other's physical neighborhood are likely to be in linkage disequilibrium and less likely to change their state (from P to N or from N to P).

Each state in the model emits a random variable *n*_A_, corresponding to the number of variant counts at a given marker site originating from the superior parent. *n*_A_ ranges from 0 to *n*, with *n* being equal to the (known) total variant count for the marker site. *n*_A_ is described by a beta binomial distribution, which allows capturing different emission probabilities in phenotype-linked versus neutral states by choosing different α and β parameters for their corresponding distributions (Figure 2B). We modeled all neutral states with the parameters α_N_ and β_N_, and all phenotype-linked states with the parameters α_P_ and β_P_. While for the neutral states α_N_ should almost equal β_N_ to make values of *n*_A_ closer to *n*/2 more likely to be sampled, for the phenotype-linked states α_P_ should be much larger than β_P_ to make values of *n*_A_ close to n more likely to be sampled.

The ratio between α_P_ and β_P_ thus defines the degree to which the relative variant frequency at a marker site needs to differ from the one obtained through random inheritance for it to be called linked to the phenotype (stringency of the method). Changing the ratio affects the probability with which an observed relative variant frequency is interpreted by the model as a phenotype linked region (see also below). In our experiments, we altered the ratio between α_P_ and β_P_ by fixing β_P_ equal to 1 and testing different values of α_P._ A cut-off on the obtained posterior probability of each marker site to be linked to the phenotype was used to prioritize the most likely causative marker sites for the phenotype of interest.

### Parameter sensitivity of EXPLoRA

We tested to what extent changing the model parameters (i.e. the α_P_/β_P_ ratio and the recombination rate) affect the results in terms of the recovery rate, the number of falsely predicted linked regions and the average size of the predicted regions. Tests were performed under two different settings that assess respectively the effect of diluting the signal to noise ratio and the resolution of the BSA. Changing signal to noise ratio’s is simulated as explained in Materials and methods (PSC) and mimics the effect of e.g. having an incomplete QTL effect of the causal genes, because for instance several minor alleles might be involved or because of an imperfect selection procedure of the segregants. The BSA resolution was altered by varying the number of marker sites in the artificial set up (see Materials and methods).

**Figure 3:**
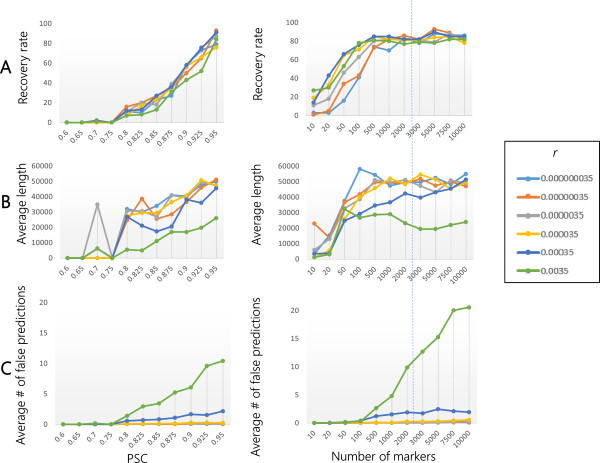
**Effect of the recombination rate (r) on the performance of EXPLoRA.** The recovery rate **(panel A)**, average size of the linked region **(panel B)** and number of falsely predicted regions **(Panel C)** as a function of the noise level (left sided plots) and the number of marker sites (right sided plots). The noise level is represented by the ratio of the segregants in the pool that have the causal allele versus those that have not (PSC). Results obtained with a number of markers that occur in real experimental settings are indicated with a dotted line.

**Figure 4:**
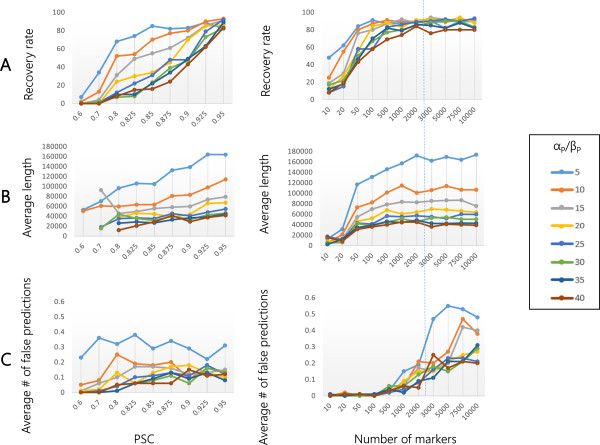
**Effect of α**_**P**_**/β**_**P **_**on the performance of EXPLoRA.** The recovery rate **(panel A)**, average size of the linked region **(panel B)** and number of falsely predicted regions **(Panel C)** as a function of the noise level represented by the ratio of the segregants in the pool that have the causal allele versus those that have not (PSC) (left sided plots) and the number of marker sites (right sided plots). Results obtained with a number of markers that occur in real experimental settings are indicated with a dotted line.

Both Figures 3 and 4, show that irrespective of the choice of the parameters, the recovery rate will drop with the noise in the dataset (noise equals lower QTL effect), the average region size becomes smaller with increasing noise levels (an observation we also made in the real data) and the number of falsely predicted linked regions is quite noise independent (except for extreme overestimations of *r*, see also below). When the signal/noise level decreases, a longer region with truly deviating relative allele frequencies (true causal site in an LD region) will have more chance to become interrupted as the distinction between signal and noise is not that clear. As EXPLoRA is designed to detect regions for which the deviating allele frequencies towards the superior allele are consistently maintained between neighbouring markers, EXPLoRA in most cases still allows detecting the region encompassing the true causal site (as here the relative allele frequencies deviate most pronouncedly) but not the regions located more towards the end of the LD region. Higher noise levels thus result in smaller identified regions without interfering with the number of falsely predicted linked regions.

Figures 3 and 4 also show that the recovery rate, the region sizes and the number of falsely predicted linked regions (except for extreme overestimations of *r*, see also below) are almost independent of the BSA resolution (the number of marker sites), provided a minimal number of markers is available. In the following, we will focus on the effect of the parameter choices on the results of EXPLoRA.

The parameter ‘recombination rate (*r*)’ determines the shape of the transition probability function which models the change from the N-state to the P-state and vice versa. EXPLoRA predicts causal sites by transitioning between these states. Gradually overestimating/underestimating the recombination rate, decreases the impact of linkage disequilibrium in modeling the effect between neighboring sites. How this affects EXPLoRA is shown in Figure 3 (both for different noise levels value and number of markers). In general, as *r* is gradually more overestimated, markers sites will be treated increasingly independent and each region with a sufficiently deviating relative allele frequency will be predicted as being linked to the phenotype, even spurious signals. This is clear in Figure 3 that shows that independent of the noise level or the number of markers (provided you have a minimal number of 1000 markers), seriously overestimating *r* results in smaller linked region sizes of the true peaks. This, however, comes at the expense of selecting a much higher number of false positive regions. Expectedly, this behavior is most pronounced under conditions with a high number of markers as under those conditions the chance of introducing spurious signals is higher. The behavior is also more present at low noise levels which is counterintuitive, but can simply be explained by the fact that at high noise levels EXPLoRA does not identify any linked regions, not even spurious ones. However, at low noise levels when regions are identified, overestimating *r* results in splitting up a truly linked region into smaller regions because the method becomes more sensitive to the small noisy variations in allele frequencies. So rather than identifying truly falsely linked regions, a high value of *r* only results in splitting up a truly linked region.

In contrast to the number of false linked regions and the region size, the recovery rate is unaffected by the choice of the parameter *r*. Contrarily to overestimating *r*, underestimating r almost does not affect the results.

Changing the α_P_/β_P_ ratio affects the emission probability or the probability with which an observed relative variant frequency is interpreted by the model as a phenotype linked region. Increasing the α_P_/β_P_ ratio makes the prediction more stringent, meaning that a higher deviation of the relative allele frequency is needed before the region is considered linked.

The results in Figure 4 are consistent with this explanation: expectedly a lower α_P_/β_P_ (less obvious relative allele frequency deviations needed) increase the recovery rate. Interestingly, the choice of α_P_/β_P_ does not affect the number of falsely linked regions (except maybe for α_P_/β_P_ = 5, but also here the number of falsely linked regions is still lower than one per dataset), but it rather affects the average size of the linked regions. This means that provided the parameter *r* is not overestimated and linkage disequilibrium is taken into account, consistency between neighboring marker sites will compensate for the spurious deviations in relative allele frequencies. Making the ratio α_P_/β_P_ less stringent will thus only extend the size of the truly linked region, but does not affect the number of false positive predictions.

Also the recovery rate, region size and the number of false positive linked regions (note the scale of the plot in this case) as a function of the number of marker sites is relatively independent of the choice of α_P_/β_P_. For a high number of markers, it seems that a less stringent α_P_/β_P_ ratio results in a relatively higher number of false positives (although again the absolute numbers are still lower than 1 false positive peak per dataset). To some extent introducing more markers will result in a higher chance of also detecting spuriously deviating relative allele frequencies.

Conclusively, at a number of available marker sites comparable to those found in real life situations (e.g. ~2 500 marker sites in 750 Kb is comparable to the yeast real data analyzed in this paper), and choosing a value for *r* that approximates the real recombination rate (which can be estimated from real data), EXPLoRA will be able to predict truly linked regions with very little false positive regions, even for experimental settings with low QTL effect (meaning that the expected relative allele frequency at the causal site is low). The choice of α_P_/β_P_ allows tuning the tradeoff between the recovery rate and the size of the linked region but does not interfere too much with the number of false positive regions.

### Comparison with state of the art

To illustrate the added value of explicitly modeling linkage disequilibrium (LD) in EXPLoRA, we ran our tool on simulated datasets and compared its performance to that obtained with the method of Magwene et al. [7] and MULTIPOOL [16]. The first one is a state-of-the-art method for the analysis of BSA results, belonging to the class of statistical methods that apply a windows-based strategy to capture the block-like behavior of the relative allele frequencies plotted along the genome. MULTIPOOL uses a dynamic Bayesian network to model the changes in relative allele frequencies along the chromosome.

Simulations mimicked different BSA experiments, differing from each other in their noise level (high and low noise level), the number of selected segregants (*n*) and the coverage (*c*) at which this pool was sequenced. Note that in our simulation set up, the noise level is mimicked by fixing the ratio of the segregants in the pool that have the causal allele versus those that have not. As a result, except for the higher impact of sampling errors at low *n*, the noise level in our simulation set up is independent of the number of selected segregants *n*.

For each experimental set up 100 different datasets were simulated and performances were assessed by the recovery rate, the false positive detection rate, and the average region size as described in Materials and methods.

**Figure 5:**
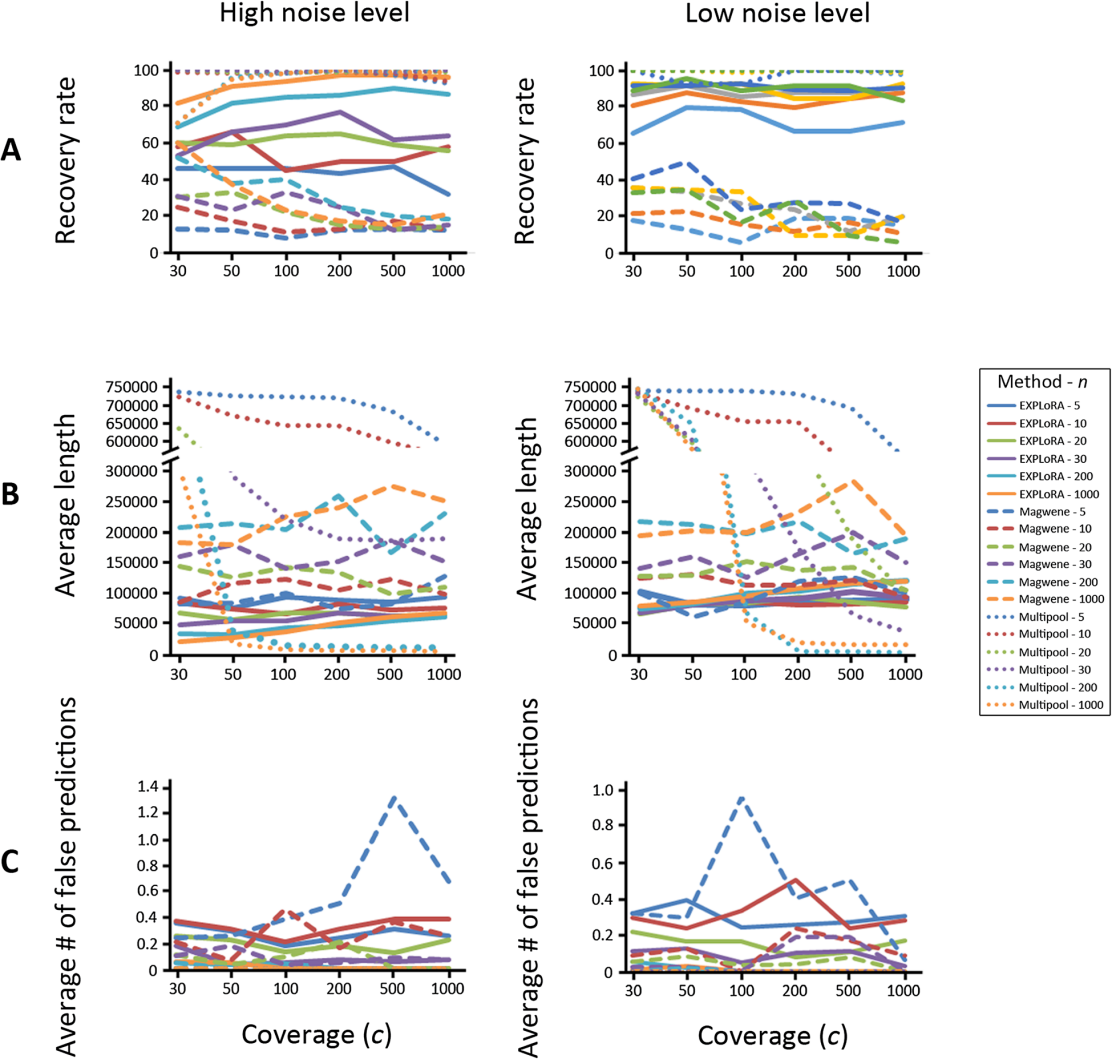
**Comparison with the state-of-the-art.** The recovery rate **(panel A)**, average size of the linked region **(panel B)** and number of falsely predicted regions **(Panel C)** under high (left sided plots) and low (right sided plots) noise levels were assessed for EXPLoRA the method of Magwene et al. and MULTIPOOL. In the plots of panel **B** (average size of the linked region) the y-axis was split into two scales to facilitate showing the results of MULTIPOOL without compressing the curves obtained by EXPLoRA and the method of Magwene et al.

Figure 5 shows that expectedly for both the method of Magwene et al. [7] and EXPLoRA the recovery rate decreases with the noise in the dataset. The number of false positives is quite noise independent for both methods. For the method of Magwene et al. [7], and for a given *n, c* combination, the size of the linked region is relatively independent of the noise level, whereas for EXPLoRA we again observed a decrease in region size with the increase in noise level (as was already noted above). For both methods the performance (recovery rate, number of falsely linked regions) decreases with a lower number of segregants. This is due to the fact that at low *n* values, sampling errors increase i.e. the relative impact of by chance including a segregant that does not carry the causal allele is higher. For the method of Magwene et al. a low *n* also interferes with the used statistics, further exacerbating the dropin performance at low *n*. This is also the reason why Magwene et al. [7] specifically recommend against applying their method on data obtained from small segregant pools.

Given the used parameter and the multiple correction settings, EXPLoRA obtains a higher recovery rate with smaller regions sizes for both noise levels than the method of Magwene et al. [7]. This low recovery rate of Magwene et al. [7] is mainly due to the stringency in the selection imposed by the robust correction for multiple testing [20] as the raw linkage scores prior to the correction were observed to be genuinely high at truly linked regions. The robust correction for multiple testing also results in the counterintuitive decrease of recovery rate of Magwene et al. [7] with increasing coverage, a behavior that was not expected based on the visual interpretation of the raw linkage score (G’) (see Additional file 1: Figure S1). Using the less stringent correction for multiple testing [24] (which does not take into account dependency between tests) compensates for this loss of recovery rate, but comes at the expense of a much larger linked regions (see Additional file 2: Figure S2).

Given its default parameter settings, MULTIPOOL [16] selects under all tested conditions (noise levels, number of segregants) one region which almost always contains the causal site, but which can be excessively large (as large as the full chromosome). As a result the recovery rate and the number of falsely predicted regions always tend to be respectively 100 and 0. Therefore the size of the detected regions is much more informative to assess the performance of MULTIPOOL [16] than recovery rate and number of falsely predicted linked regions. Given a sufficiently high number of segregants *n* and a minimal coverage *c*, MULTIPOOL outperforms EXPLoRA in better estimating the region close to the true causal site. However, compared to EXPLoRA, MULTIPOOL [16] is less robust to changes in the number of segregants (*n*) and the coverage (*c*) than EXPLORA and it starts underperforming compared to EXPLORA in the presence of few segregants and low coverage. This is because in contrast to EXPLoRA, which estimates the transition probability to move from a linked to a non-linked state from a negative exponential model as a function of the recombination rate *r* and the physical distance between neighboring marker sites, MULTIPOOL [16] uses the change in the estimates of the relative allele frequencies between neighboring marker sites to calculate the transition probability. When the number of segregants (*n*) is small or the coverage (*c*) is low, there are insufficient data to correctly estimate the distribution of the relative allele frequencies along the chromosome correctly and thus to obtain correct estimates of the transition probability. As a result, chances are higher of obtaining transitions probabilities close to 0 across neighboring marker sites, which to our opinion explains why MULTIPOOL [16] outputs very long linked regions at a low number of segregants.

Conclusively, EXPLoRA shows state-of-the-art performance. More importantly its performance remains extremely robust even when lowering the number of selected segregants or when the signal/noise level is low. These properties make the method particularly useful under BSA conditions for which segregant selection is non-trivial or the QTL effect is minor (e.g. when several minor alleles are contributing to the phenotype).

### Application of EXPLoRA to real datasets

To evaluate the performance of our analysis method with a real BSA experiment, we applied EXPLoRA to the data described in Swinnen et al. [3]. In their analysis they used a statistical smoother to facilitate detecting from the raw data regions with deviations in relative allele frequencies. Based on visual inspection and comparing the results from the 16 and 17% pool allowed them to predict six loci as being significantly linked to the phenotype, all of which were also explicitly mentioned in the paper. Of those loci, the ones located on chromosomes V, X and XIV were denoted as respectively QTL1, 2 and 3 by Swinnen et al. The three remaining loci, located on chromosomes II, XII and XV did not receive a QTL number in the publication by Swinnen et al. In the original paper, QTLs 1, 2 and 3 were further proven to be statistically linked by individual genotyping of SNP markers surrounding each QTL [3].

**Figure 6:**
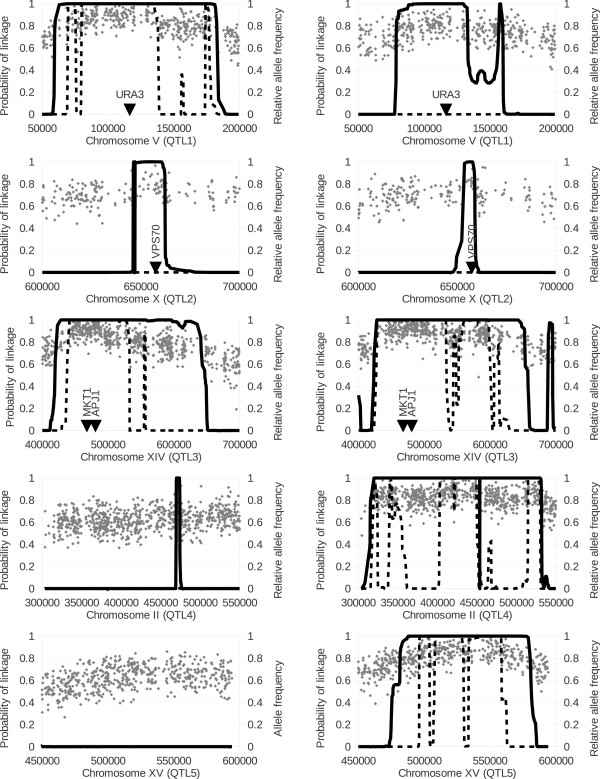
**Linkage scores obtained by EXPLoRA for the five QTLs identified in the 16% pool (left) and in the 17% pool (right).** The original relative variant frequencies as determined by genome sequencing are displayed for each plot (light gray dots). Solid lines show the posterior probabilities for α_P_/β_P_ = 10 whereas dashed lines show the posterior probabilities for α_P_/β_P_ = 30.

To test to what extent we could recapitulate their results, we ran EXPLoRA with both α_P_/β_P_ = 30 and α_P_/β_P_ = 10 ratios and a cut off on the posterior probability score of 0.95 on the pools selected for 16 and 17% ethanol separately. In Figure 6 the most confident results are shown i.e. those results that either could be confirmed with both parameter settings (the most and the least stringent that is α_P_/β_P_ = 30 and α_P_/β_P_ = 10) or that could be confirmed in both pools (16 and 17% ethanol) with at least one parameter setting. With α_P_/β_P_ = 10 and setting a minimum posterior probability of linkage of 0.95 we predicted in the 16% pool 923 marker sites clustered in four loci. In agreement with the initial study of Swinnen et al. [3] we identified the experimentally verified QTL1 located on chromosome V between coordinates 116,000 and 117,000, containing the causative gene *URA3*. QTL2 located on chromosome X between coordinates 646,155 and 662,146 (for which no causative gene was reported in the original work of Swinnen et al. [3]) and QTL3 encompassing a gene cluster on chromosome XIV between coordinates 466,000 and 486,000, containing the causative genes *MKT1* and *APJ1*. In addition, we detected one locus that was mentioned, but not further validated in the initial publication: a small, but still significant region on chromosome II (referred to in this study as QTL4 encompassing 18 of the marker sites (Figure 6)). The length of the linked regions identified with α_P_/β_P_ = 10 varies from as small as 4.3 kbp for QTL4 to as large as 226 kbp in QTL3.

These four QTLs (QTL1, 2, 3, and 4) identified in the 16% pool were also detected in the analysis of the 17% ethanol pool using EXPLoRA with the same parameter settings (α_P_/β_P_ =10), further increasing the confidence that these QTLs were truly linked to ethanol tolerance (these regions encompassed a 757 (37.2%) of the total number of linked marker sites (2,034) in the 17% pool). In addition the more stringently phenotypic selection of the 17% pool allowed drastically decreasing the length of QTL1 and QTL2 (reducing them from 123 kbp and 16 kbp to 58 kbp and 5.3 kbp respectively) as detected by EXPLoRA with α_P_/β_P_ =10.

The remaining 607 linked markers in the 17% pool mapped to a locus (that was mentioned, but not further validated in the initial publication) encompassing a region of 105 kbp in chromosome XV (referred to in this study as QTL5) and to three small regions on chromosomes I, VI, and XII (of which the latter one is also mentioned in the initial publication but not further validated). Neither of those QTLs was detected in the 16% pool, indicating that they are specifically enriched at more extreme ethanol levels (17%). The fact that the region at chromosome XV (QTL5) could also be confirmed with the more stringent value of α_P_/β_P_ = 30 (see also below) indicates that from these additional QTLs, this region is the best candidate to be an additional truly linked region. Using the same settings (α_P_/β_P_ = 10 and α_P_/β_P_ = 30 and a cut off on the posterior probability score of 0.95), EXPLoRA did not report significant relationship with ethanol tolerance for any polymorphic site in the control pool of unselected segregants.

Figure 6 further illustrates the effect of changing the α_P_/β_P_ ratio on the recovery rate and the size of the linked region for the identified QTLs on respectively the 16 and 17% pool. As predicted by the simulation experiments, changing the ratio α_P_/β_P_ from less (10, solid line) to more stringent values (30; dashed lines) reduces the length of the linked region size, but comes at the expense of missing the least pronounced QTLs. For instance, for the 16% ethanol pool increasing the α_P_/β_P_ ratio, reduces the length of QTLs from 123 kbp to 66 kbp and from 226 kbp to 93 kbp in QTL1 and QTL3 respectively. However, this more stringent setting results in missing QTL2 and QTL4 (dashed lines in Figure 6) in the 16% pool, indicating that for this pool the signals of these QTLs are not very pronounced (minor QTLs in 16% ethanol). Equally, in the 17% pool increasing the stringency of EXPLoRA, reduces the length of the linked regions in QTL3, 4 and 5, but results in missing QTL1 and QTL2 and the additional smaller linked regions in chromosomes I, VI, and XII.

These results indicate that the signal of QTL3 is prominent in both pools and thus very relevant for ethanol tolerance under both ethanol conditions. The signal of QTL1 is clearly more pronounced in the 16% pool than in the 17% pool, whereas for the signals of QTL4 and QTL5 the opposite is true, implying that under both ethanol conditions other protection mechanisms tend to play a role. The region in QTL2, despite being a minor locus (not such pronounced signal) might play an equally important role under both ethanol conditions as it is recovered in both pools.

### Experimental validation of the newly predicted QTL2 on chromosome X

**Figure 7:**
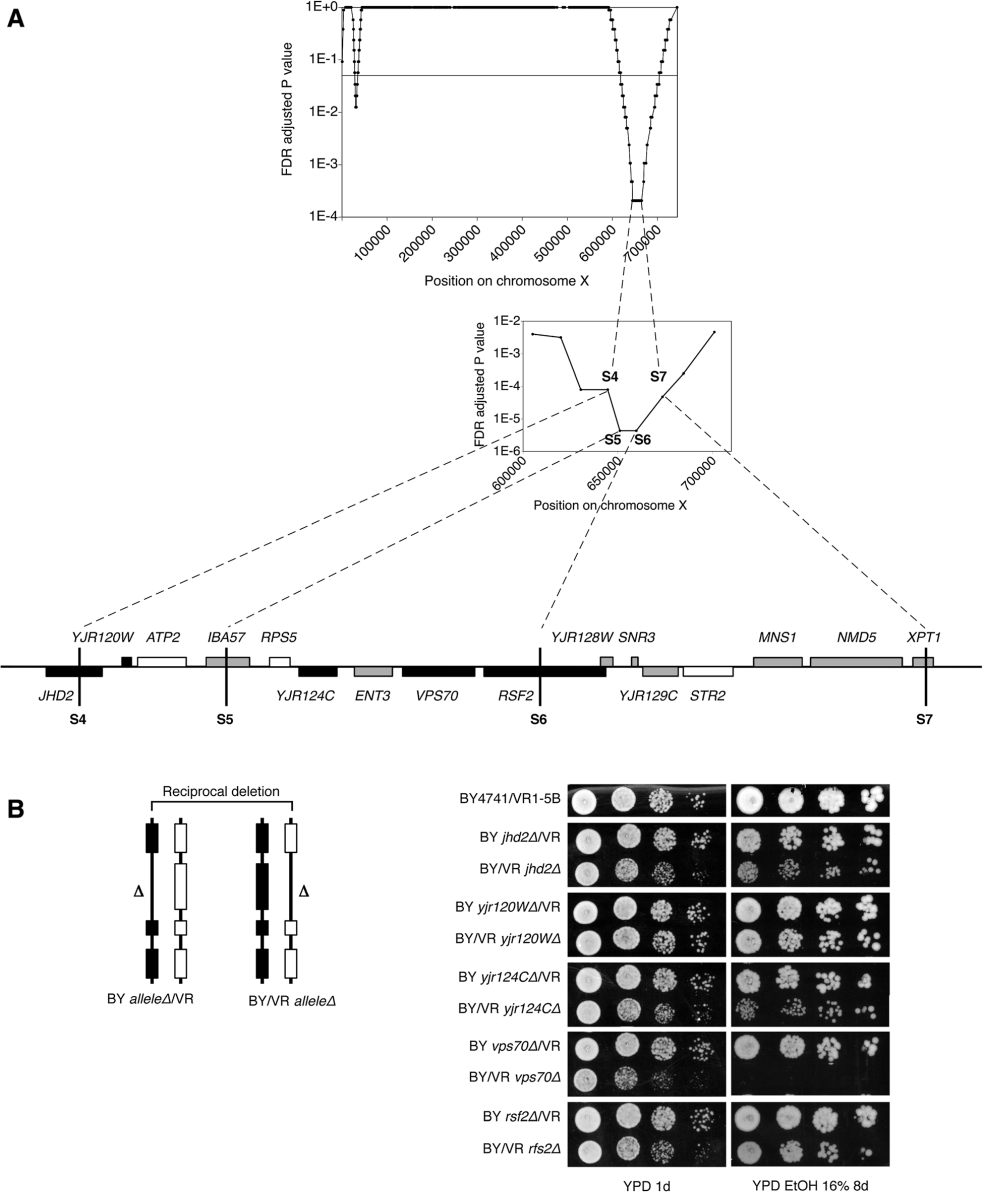
**Experimental validation of QTL2 on chromosome X. A**: upper plot shows the region corresponding to QTL2 of which linkage to the phenotype of interest was confirmed by scoring selected marker sites in individual segregants. Scored marker sites are indicated (S4-S7). For each marker site, the p-value indicates the probability to be linked to the phenotype by chance according to a binomial distribution (see materials and methods). Lower plot: zoom in on the genes in the experimentally confirmed region corresponding to QTL2 (29 kb). Black bars: genes with non-synonymous mutations in the coding region; grey bars: genes with mutations in the promotor or terminator; white bars: genes without mutations. **B**: Reciprocal hemizygosity analysis for the genes with non-synonymous mutations in the coding regions located in the fine-mapped region. To that end, two different diploid strains were constructed by crossing the original superior parent VR1-5B with the inferior parent BY4741, carrying a deletion in its allele of the candidate causative gene or the other way around. Hence, this resulted in two different diploid strains, each with only one functional allele of the candidate causative gene, originating from either the ‘superior’ or the ‘inferior’ parent. The ethanol tolerance of the two diploid strains was compared with dilution spot growth assays on a YPD plate with 16% ethanol and a YPD plate without ethanol as control.

To assess the validity of our predictions, we selected QTL2 (on chromosome X) for experimental validation as this QTL, despite being important in both the 16% and 17% pool seemed to be one of the more difficult QTLs to detect (only confirmed by the least stringent selection criteria). Fine-mapping of the region by PCR-based scoring of the markers in the individual segregants (Materials and methods), allowed us to confirm the area with the strongest link. Mutations in this confirmed region were verified by Sanger sequencing. All genes carrying non-synonymous mutations in their coding region were first selected as candidate causative genes (Figure 7A). True causative genes in QTL2 were identified using reciprocal hemizygosity analysis [25]. For each candidate causative gene a set of two diploid strains was constructed by crossing the parental strains, either containing or lacking the candidate gene. As a result each diploid has a different allele of the candidate gene while the other copy of the gene is deleted (Figure 7B). Phenotypic analysis on YPD plates with 16% ethanol showed a clear difference in ethanol tolerance between the two diploid strains carrying a different allele of *VPS70:* the strain with the allele derived from the VR1-5B superior parent grew very well in the presence of 16% ethanol, whereas the strain with the allele from the BY4741 inferior parent did not grow at all (Figure 7B), indicating that *VPS70* carries a causative mutation responsible for the link of QTL2 with high ethanol tolerance. Except for a putative role in sorting of vacuolar carboxypeptidase Y to the vacuole [26], no link to ethanol tolerance for *VPS70* has been reported previously. This may be due to the fact that all previous analyses of yeast ethanol tolerance were performed with laboratory strains and with much lower ethanol concentrations (e.g. [27]).

## Discussion

In contrast to previously applied single locus models [3,4], most state-of-the-art methods to analyse the results of BSA exploit the dependencies between neighbouring sites to better distinguish truly from spuriously linked regions. Whereas classical data-driven statistical approaches fit a complex smoothing function to the data to facilitate the identification of patterns in the relative variant frequency plots, EXPLoRA explicitly models linkage disequilibrium to explain the observed patterns in the data, which allows to compensate for noise caused by sampling and sequencing errors, and for the low statistical power in case of small pools or incomplete QTL effects. This was clearly illustrated in the simulation experiments where under conditions that become restrictive for a state-of-the-art statistical method such as the one of Magwene et al. EXPLoRA was still able achieve a high recovery rate while keeping a permissible low number of falsely linked regions.

A similar philosophy as the one adopted by EXPLoRA is also used in the recently published methods MULTIPOOL [16] and the model of Leshchiner et al. [14]. MULTIPOOL [16] uses a Bayesian network to explictly infer variation in allele frequencies along the chromosome. Such approach allows to better define the region close to the true causal site, but comes at the expense of having to estimate more parameters in the model, which becomes restrictive at low coverage or in the presence of a low number of segregants. Results obtained with EXPLoRA on simulated data show that the specific way in which EXPLoRA models the effect of LD results in efficiently identifying phenotype-linked regions, even at low signal/noise ratio’s. These results were confirmed by reanalyzing a real dataset in which EXPLoRA was indeed able to detect additional QTLs in the 17% pool that were confirmed by the 16% pool despite the much lower number of segregants in this 17% pool. It was also able to recover for both pools a minor allele (in QTL2) for which the true contribution to ethanol tolerance was confirmed by experimentally identifying its causal gene.

## Conclusions

By using linkage disequilibrium to model the dependency between neighboring marker sites, EXPLoRA allows to reliably detect QTLs using bulk-segregant whole genome sequencing data. Results obtained with both simulated and experimental data show that EXPLoRA displays superior performance under conditions with a low signal to noise level (e.g. small selected pool size, sampling errors, incomplete QTL effects e.g. by the contribution of multiple minor alleles).

### Availability of supporting data

The sequencing data sets supporting the results of this article are available in the NCBI Sequence Read Archive (SRA) (http://trace.ncbi.nlm.nih.gov/Traces/sra/sra.cgi) under the accession number SRA049724.

## Competing interests

The authors declare that they have no competing interests.

## Authors’ contributions

ASR conceived the study. JD and ASR designed and implemented the method. ASR, JD and SPT performed the computational analysis. AG, GH, MFM and JT performed the molecular-genetic studies. JD and ASR drafted the manuscript. KM participated in the design of the computational analysis and drafting the manuscript. JT, KJV and KM coordinated and managed the research. All authors contributed to writing the manuscript and approved its final version.

## Authors’ information

Jorge Duitama, Aminael Sánchez-Rodríguez and Annelies Goovaerts are joint first Authors.

## Supplementary Material

Additional file 1: Figure S1 Average scaled linkage score at the causal site reported by the method of Magwene et al. [7] as a function of the coverage and under high **(panel A)** and low **(panel B)** noise levels. Raw values of the G’ statistics at the causal site (G’_causal_) were scaled taking into the maximum (G’_max_) and minimum (G’_min_) G’s values from the entire artificial chromosome according to the following formula: G’_scaled_ = (G’_causal_ – G’_min_)/(G’_max_ – G’_min_). Reported values correspond to the average of 100 repetitions. Click here for file

Additional file 2: Figure S2 Comparison with the state-of-the-art. The recovery rate **(panel A)**, average size of the linked region **(panel B)** and number of falsely predicted regions **(Panel C)** under high (left sided plots) and low (right sided plots) noise levels were assessed for EXPLoRA and the method of Magwene et al. [7]. For the method of Magwene et al. [7] the less stringent correction for multiple testing, which does not take into account dependency between tests, was used. Click here for file

## References

[B1] Liti G Schacherer J The rise of yeast population genomics Comptes Rendus Biol 2011 15 8–9 612 619 10.1016/j.crvi.2011.05.009 21819942

[B2] Swinnen S Thevelein JM Nevoigt E Genetic mapping of quantitative phenotypic traits in Saccharomyces cerevisiae FEMS Yeast Res 2012 15 2 215 227 10.1111/j.1567-1364.2011.00777.x 22150948

[B3] Swinnen S Schaerlaekens K Pais T Claesen J Hubmann G Yang Y Demeke M Foulquie-Moreno MR Goovaerts A Souvereyns K Clement L Dumortier F Thevelein JM Identification of novel causative genes determining the complex trait of high ethanol tolerance in yeast using pooled-segregant whole-genome sequence analysis Genome Res 2012 15 5 975 984 10.1101/gr.131698.111 22399573 PMC3337442

[B4] Birkeland SR Jin N Ozdemir AC Lyons RH Jr Weisman LS Wilson TE Discovery of mutations in Saccharomyces cerevisiae by pooled linkage analysis and whole-genome sequencing Genetics 2010 15 4 1127 1137 10.1534/genetics.110.123232 20923977 PMC2998298

[B5] Wenger JW Schwartz K Sherlock G Bulk segregant analysis by high-throughput sequencing reveals a novel xylose utilization gene from Saccharomyces cerevisiae PLoS Genet 2010 15 5 e1000942 10.1371/journal.pgen.1000942 20485559 PMC2869308

[B6] Parts L Cubillos FA Warringer J Jain K Salinas F Bumpstead SJ Molin M Zia A Simpson JT Quail MA Moses A Louis EJ Durbin R Liti G Revealing the genetic structure of a trait by sequencing a population under selection Genome Res 2011 15 7 1131 1138 10.1101/gr.116731.110 21422276 PMC3129255

[B7] Magwene PM Willis JH Kelly JK The statistics of bulk segregant analysis using next generation sequencing PLoS Comput Biol 2011 15 11 e1002255 10.1371/journal.pcbi.1002255 22072954 PMC3207950

[B8] Cubillos FA Billi E Zorgo E Parts L Fargier P Omholt S Blomberg A Warringer J Louis EJ Liti G Assessing the complex architecture of polygenic traits in diverged yeast populations Mol Ecol 2011 15 7 1401 1413 10.1111/j.1365-294X.2011.05005.x 21261765

[B9] Ehrenreich IM Torabi N Jia Y Kent J Martis S Shapiro JA Gresham D Caudy AA Kruglyak L Dissection of genetically complex traits with extremely large pools of yeast segregants Nature 2010 15 7291 1039 1042 10.1038/nature08923 20393561 PMC2862354

[B10] Quarrie SA Lazić-Jančić V Kovačević D Steed A Pekić S Bulk segregant analysis with molecular markers and its use for improving drought resistance in maize J Exp Bot 1999 15 337 1299 1306 10.1093/jxb/50.337.1299

[B11] Schneeberger K Ossowski S Lanz C Juul T Petersen AH Nielsen KL Jorgensen JE Weigel D Andersen SU SHOREmap: simultaneous mapping and mutation identification by deep sequencing Nat Methods 2009 15 8 550 551 10.1038/nmeth0809-550 19644454

[B12] Austin RS Vidaurre D Stamatiou G Breit R Provart NJ Bonetta D Zhang J Fung P Gong Y Wang PW McCourt P Guttman DS Next-generation mapping of Arabidopsis genes Plant J 2011 15 4 715 725 10.1111/j.1365-313X.2011.04619.x 21518053

[B13] Abe A Kosugi S Yoshida K Natsume S Takagi H Kanzaki H Matsumura H Yoshida K Mitsuoka C Tamiru M Innan H Cano L Kamoun S Terauchi R Genome sequencing reveals agronomically important loci in rice using MutMap Nat Biotechnol 2012 15 2 174 178 10.1038/nbt.2095 22267009

[B14] Leshchiner I Alexa K Kelsey P Adzhubei I Austin-Tse CA Cooney JD Anderson H King MJ Stottmann RW Garnaas MK Ha S Drummond IA Paw BH North TE Beier DR Goessling W Sunyaev SR Mutation mapping and identification by whole-genome sequencing Genome Res 2012 15 8 1541 1548 10.1101/gr.135541.111 22555591 PMC3409267

[B15] Hill W Robertson A Linkage disequilibrium in finite populations Theor Appl Genet 1968 15 6 226 231 10.1007/BF01245622 24442307

[B16] Edwards MD Gifford DK High-resolution genetic mapping with pooled sequencing BMC Bioinformatics 2012 15 Suppl 6 S8 22537047 10.1186/1471-2105-13-S6-S8 PMC3358661

[B17] Ruderfer DM Pratt SC Seidel HS Kruglyak L Population genomic analysis of outcrossing and recombination in yeast Nat Genet 2006 15 9 1077 1081 10.1038/ng1859 16892060

[B18] Cherry JM Ball C Weng S Juvik G Schmidt R Adler C Dunn B Dwight S Riles L Mortimer RK Botstein D Genetic and physical maps of Saccharomyces cerevisiae Nature 1997 15 6632 Suppl 67 73 9169866 PMC3057085

[B19] Glenn TC Field guide to next-generation DNA sequencers Mol Ecol Res 2011 15 5 759 769 10.1111/j.1755-0998.2011.03024.x 21592312

[B20] Benjamini Y Yekutieli D Quantitative trait Loci analysis using the false discovery rate Genetics 2005 15 2 783 790 10.1534/genetics.104.036699 15956674 PMC1456787

[B21] Langmead B Salzberg SL Fast gapped-read alignment with Bowtie 2 Nat Methods 2012 15 4 357 359 10.1038/nmeth.1923 22388286 PMC3322381

[B22] Duitama J Srivastava PK Măndoiu II Towards accurate detection and genotyping of expressed variants from whole transcriptome sequencing data BMC Genomics 2012 15 Suppl 2 S6 10.1186/1471-2164-13-S2-S6 22537301 PMC3394419

[B23] Abyzov A Urban AE Snyder M Gerstein M CNVnator: an approach to discover, genotype, and characterize typical and atypical CNVs from family and population genome sequencing Genome Res 2011 15 6 974 984 10.1101/gr.114876.110 21324876 PMC3106330

[B24] Benjamini Y Hochberg Y Controlling the false discovery rate: a practical and powerful approach to multiple testing J Royal Stat Soc Series B (Methodological) 1995 15 289 300

[B25] Steinmetz LM Sinha H Richards DR Spiegelman JI Oefner PJ McCusker JH Davis RW Dissecting the architecture of a quantitative trait locus in yeast Nature 2002 15 6878 326 330 10.1038/416326a 11907579

[B26] Bonangelino CJ Chavez EM Bonifacino JS Genomic screen for vacuolar protein sorting genes in Saccharomyces cerevisiae Mol Biol Cell 2002 15 7 2486 2501 10.1091/mbc.02-01-0005 12134085 PMC117329

[B27] Van Voorst F Houghton-Larsen J Jonson L Kielland-Brandt MC Brandt A Genome-wide identification of genes required for growth of Saccharomyces cerevisiae under ethanol stress Yeast 2006 15 5 351 359 10.1002/yea.1359 16598687

