# Supplementary figures and images for: Improved linkage analysis of Quantitative Trait Loci using bulk segregants unveils a novel determinant of high ethanol tolerance in yeast
Source: BMC Genomics. 2014 Mar 19;15:207. doi: 10.1186/1471-2164-15-207 (PMC4003806; doi:10.1186/1471-2164-15-207)

High Noise level

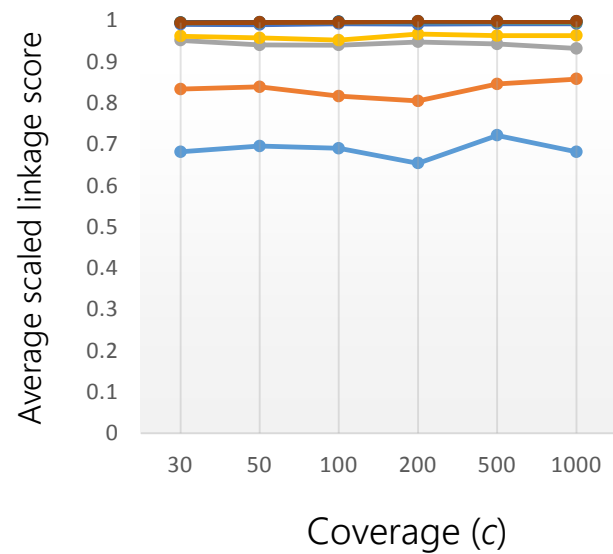

Low Noise level

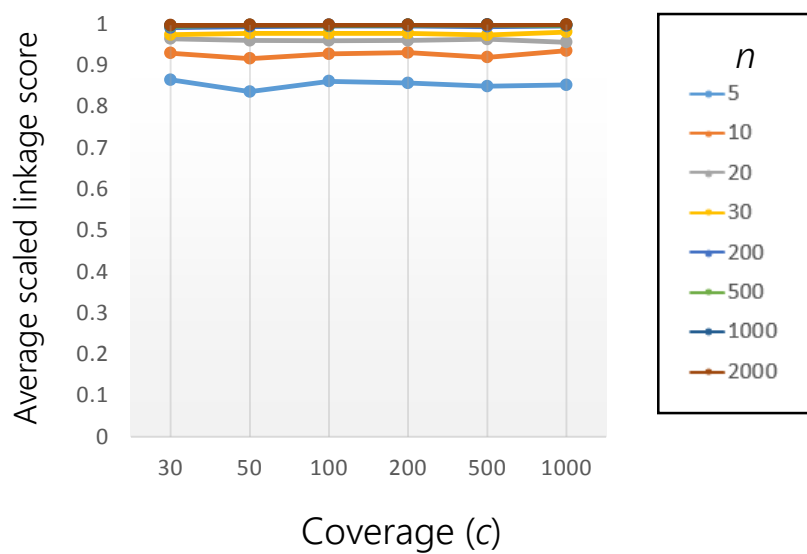

Supplement: Additional file 1: Figure S1 — Average scaled linkage score at the causal site reported by the method of Magwene et al. [7] as a function of the coverage and under high (panel A) and low (panel B) noise levels. Raw values of the G’ statistics at the causal site (G’causal) were scaled taking into the maximum (G’max) and minimum (G’min) G’s values from the entire artificial chromosome according to the following formula: G’scaled = (G’causal – G’min)/(G’max – G’min). Reported values correspond to the average of 100 repetitions. [file 1471-2164-15-207-S1.pdf]

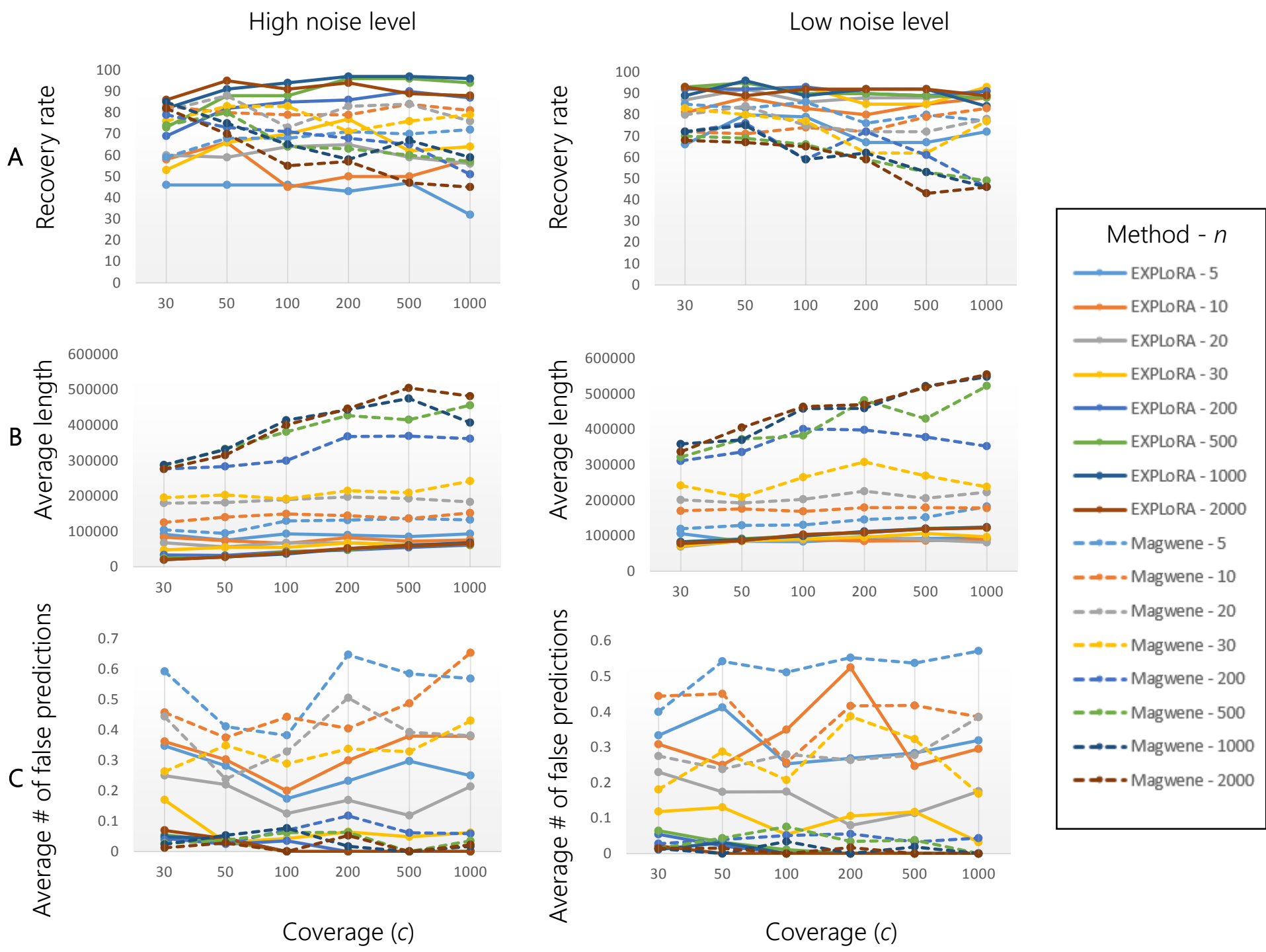

Supplement: Additional file 2: Figure S2 — Comparison with the state-of-the-art. The recovery rate (panel A), average size of the linked region (panel B) and number of falsely predicted regions (Panel C) under high (left sided plots) and low (right sided plots) noise levels were assessed for EXPLoRA and the method of Magwene et al. [7]. For the method of Magwene et al. [7] the less stringent correction for multiple testing, which does not take into account dependency between tests, was used. [file 1471-2164-15-207-S2.pdf]
